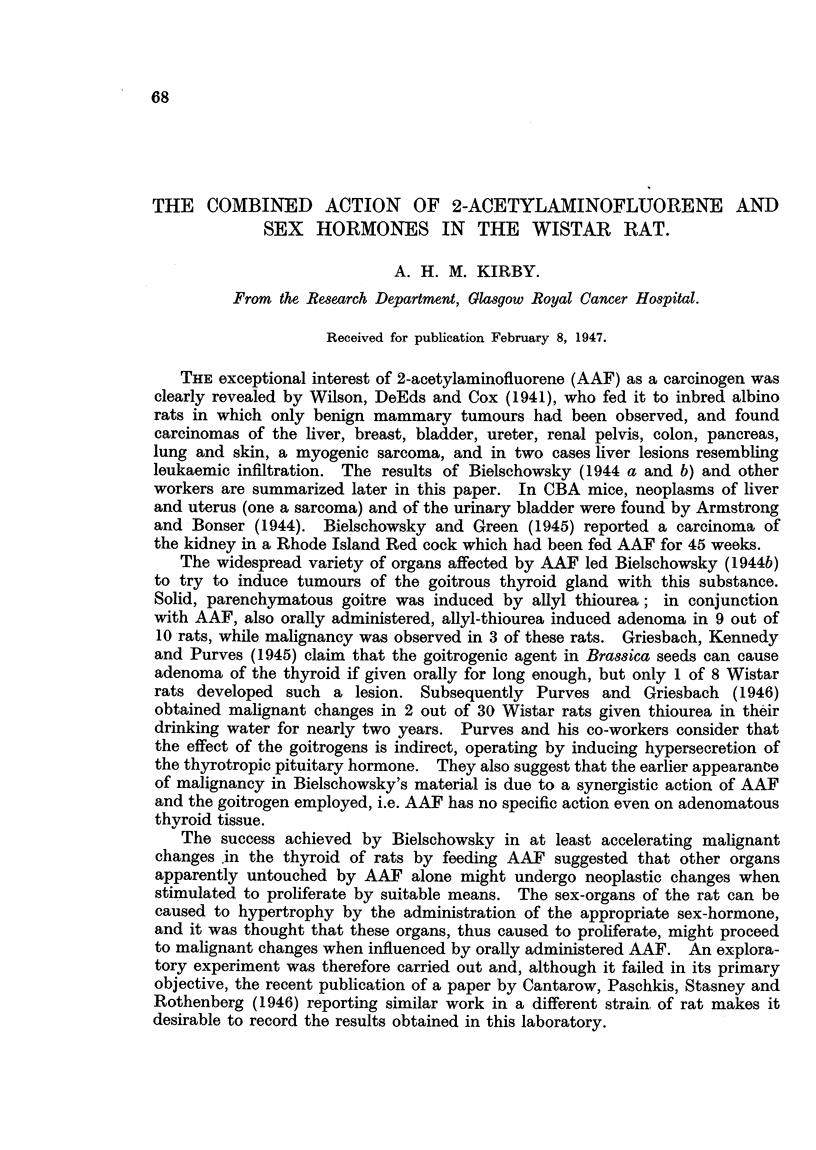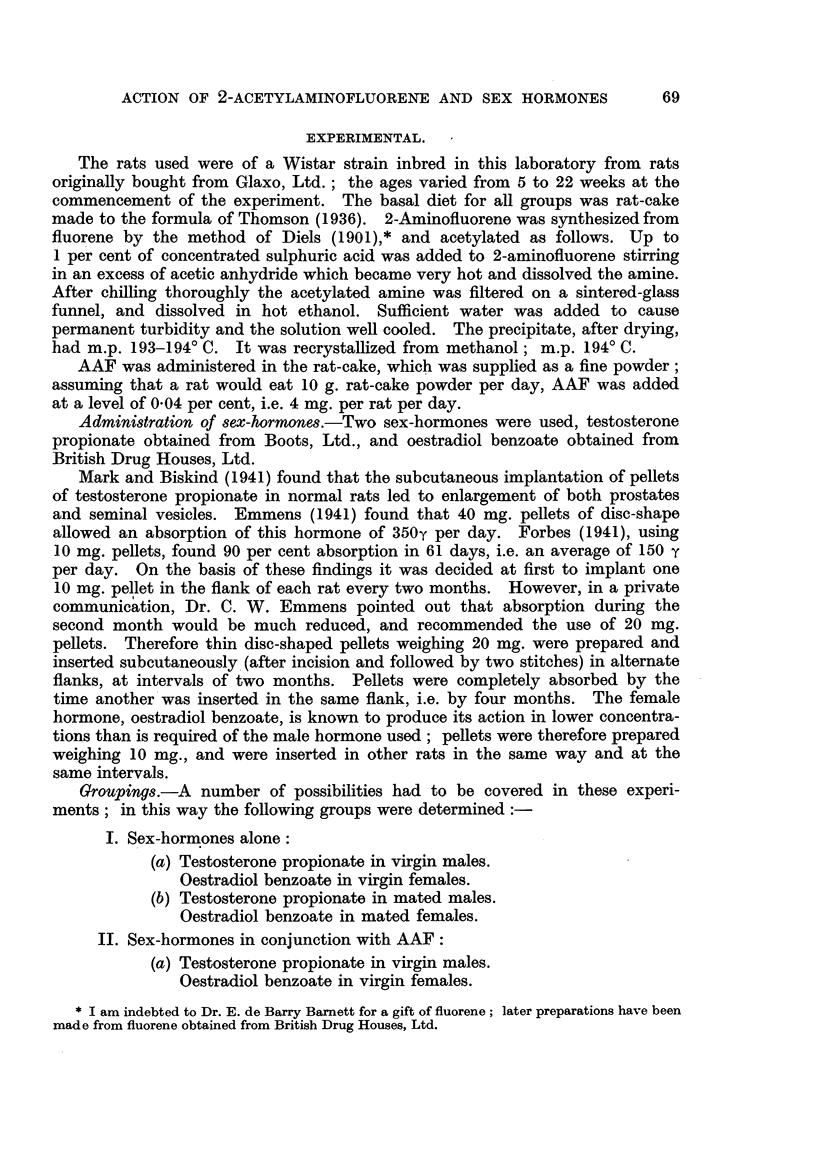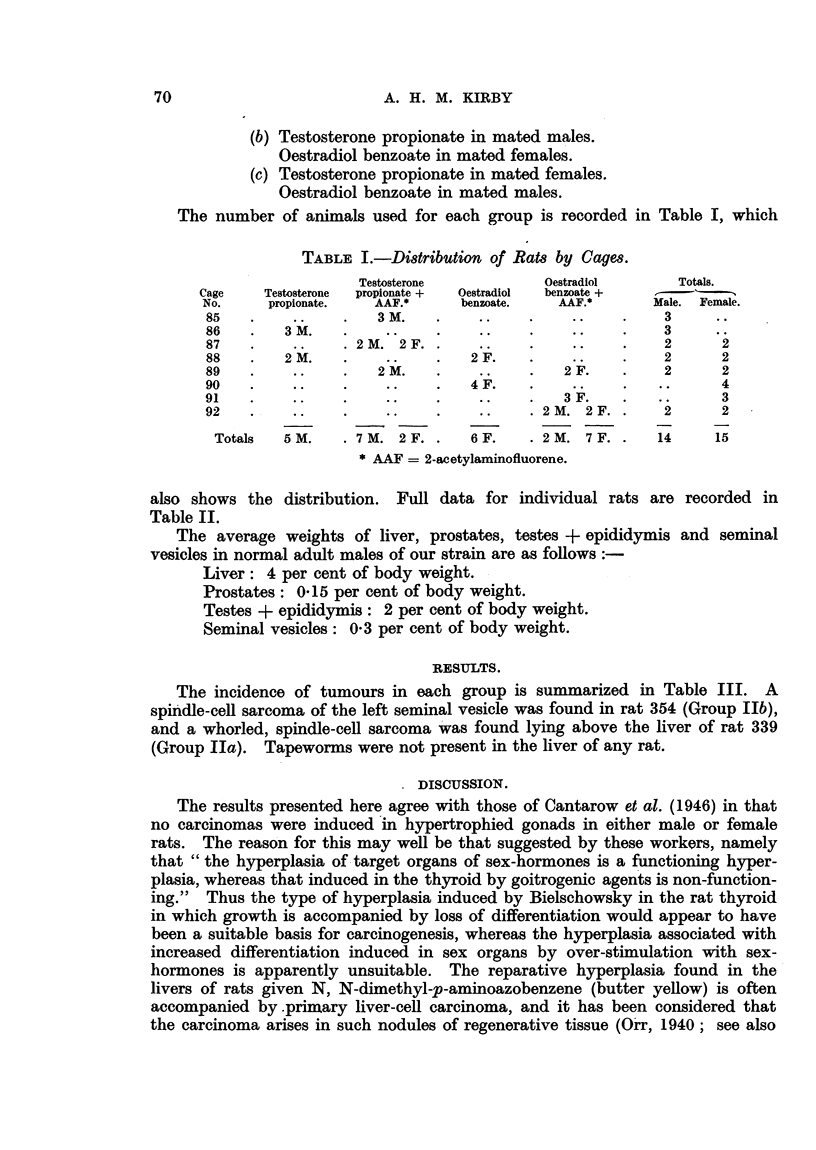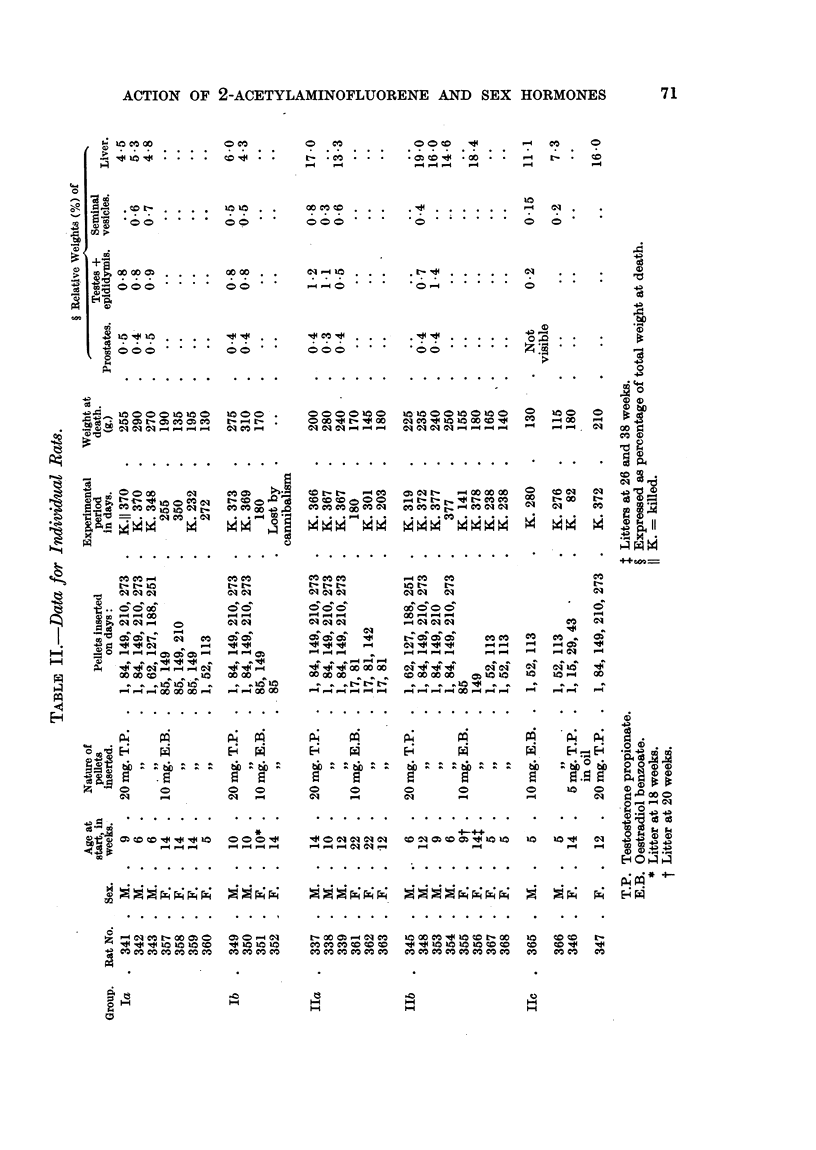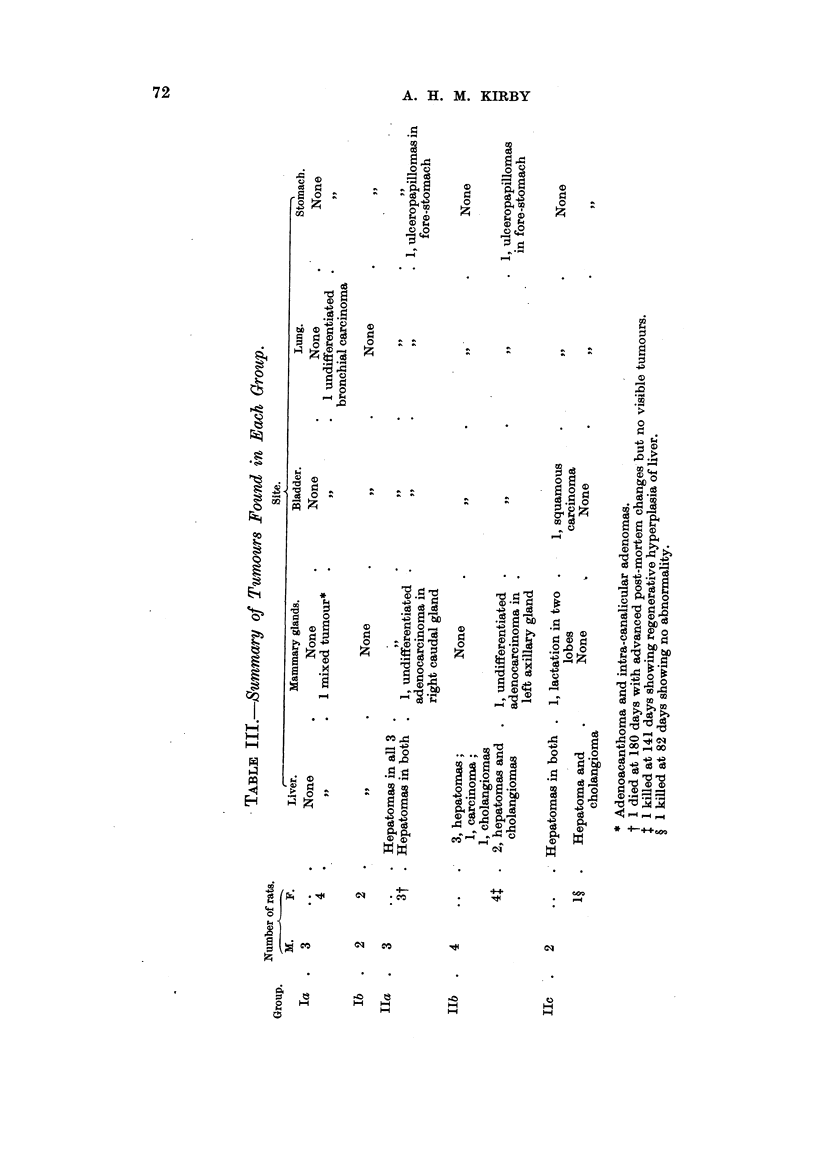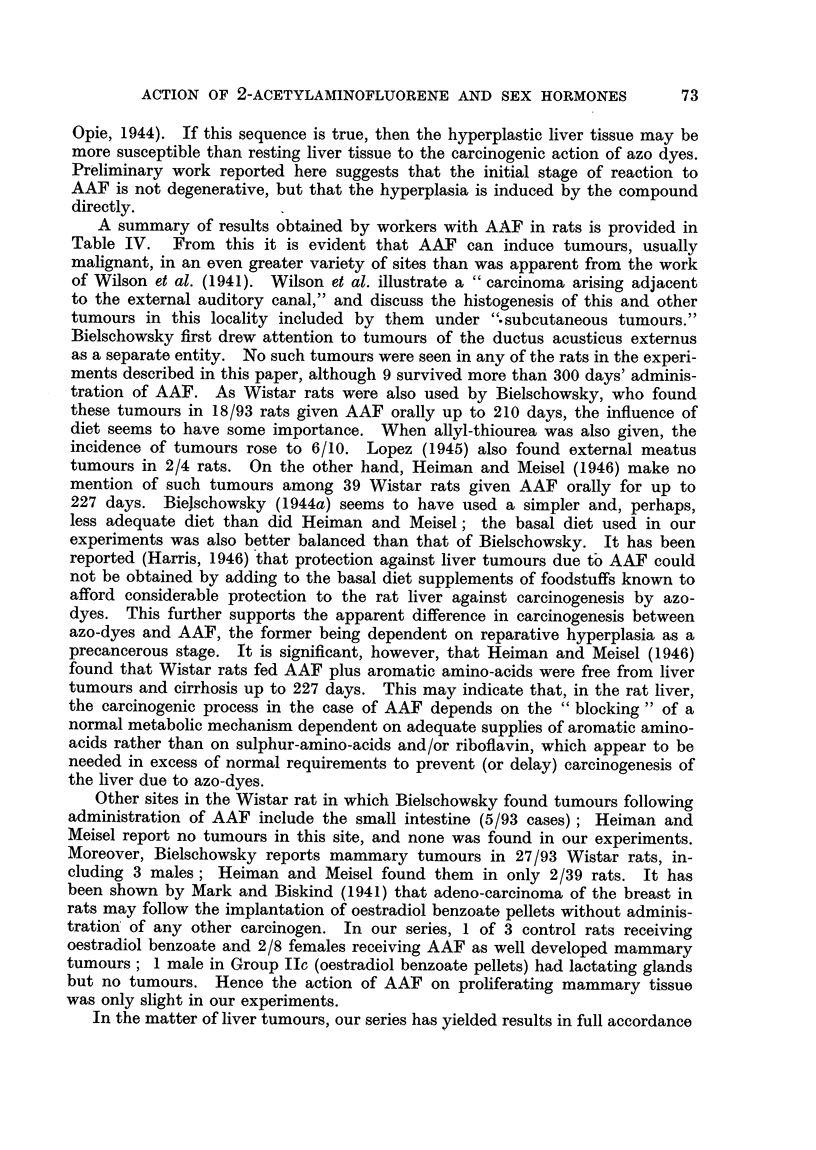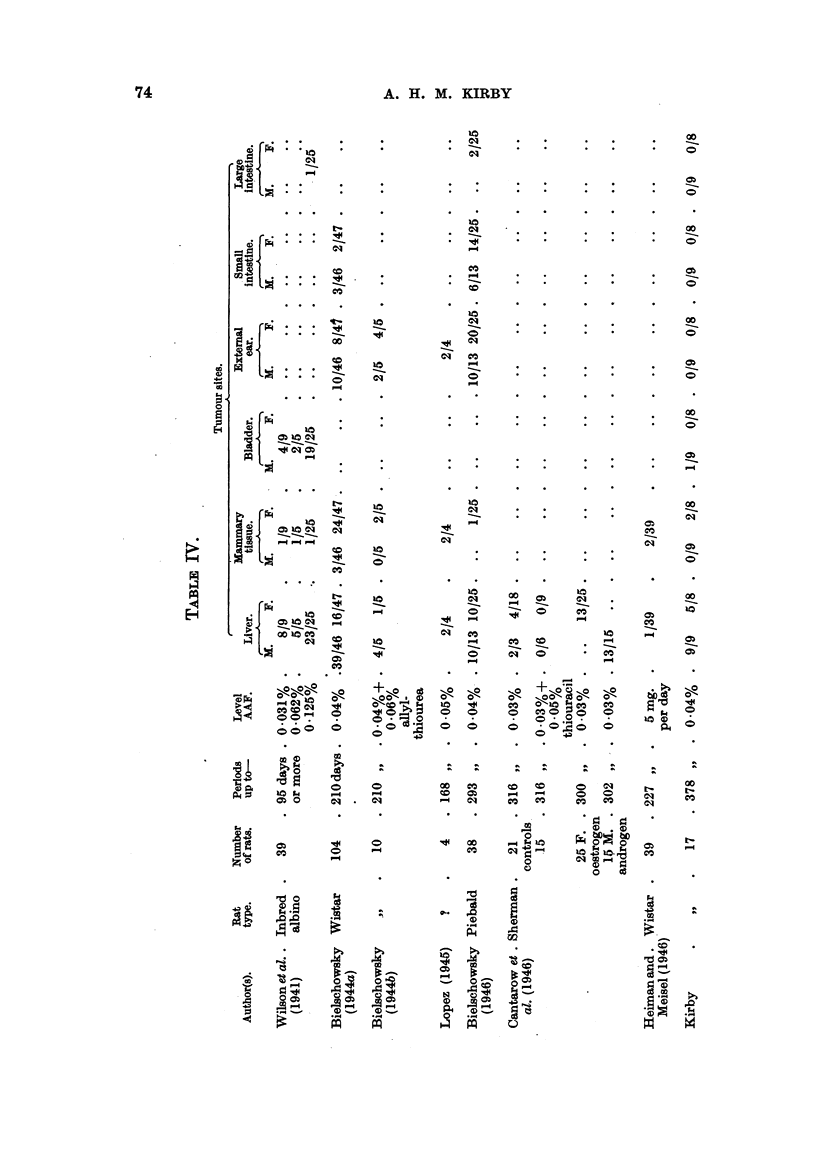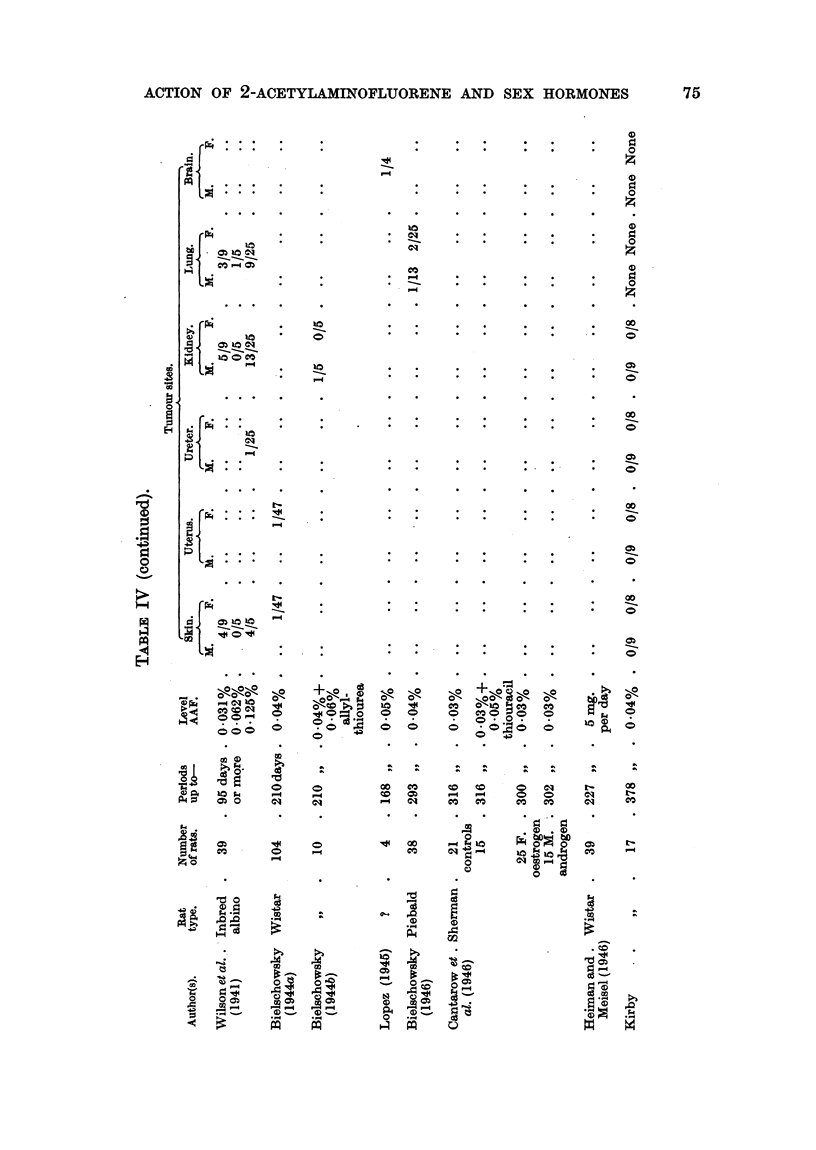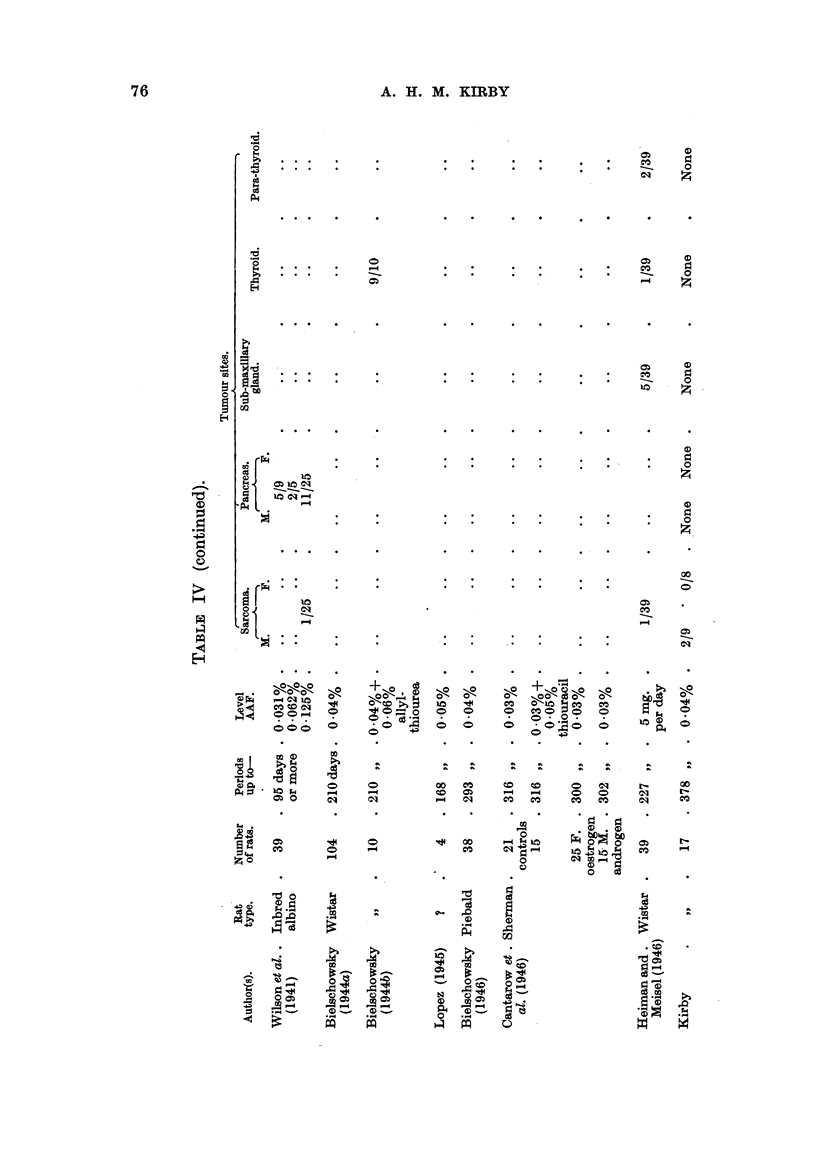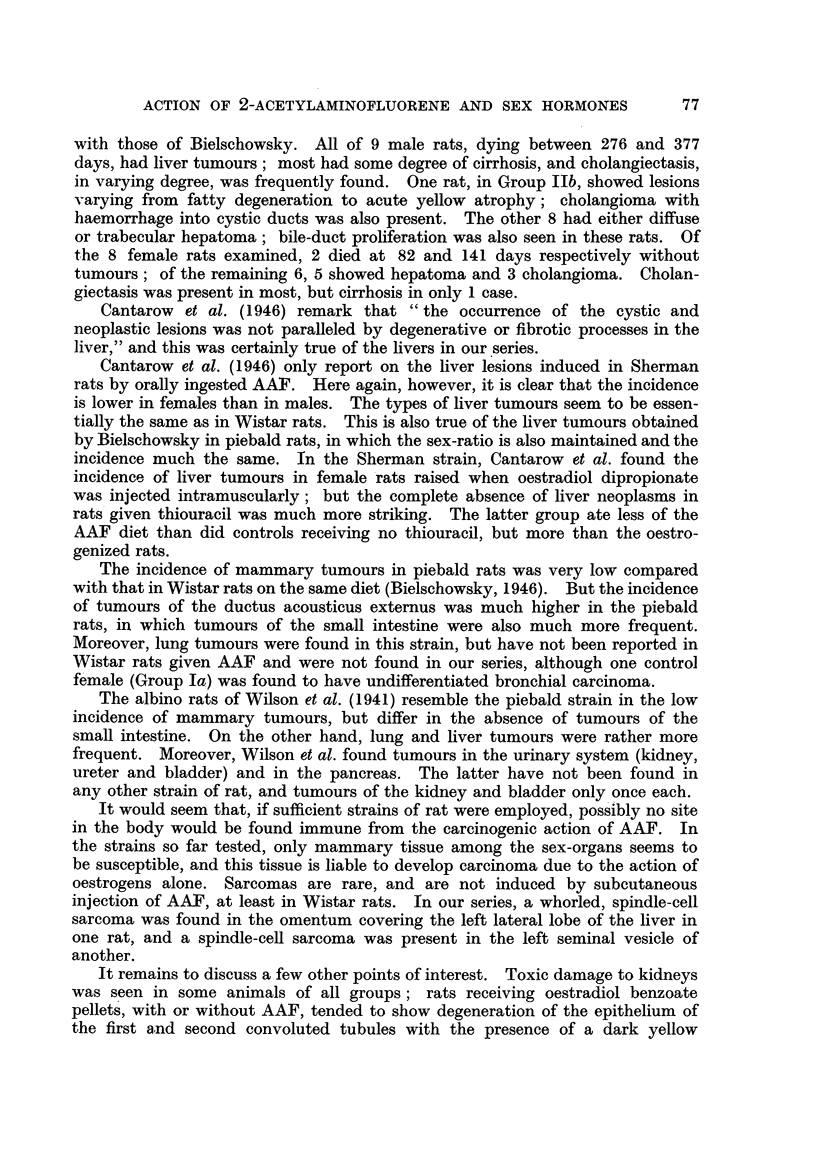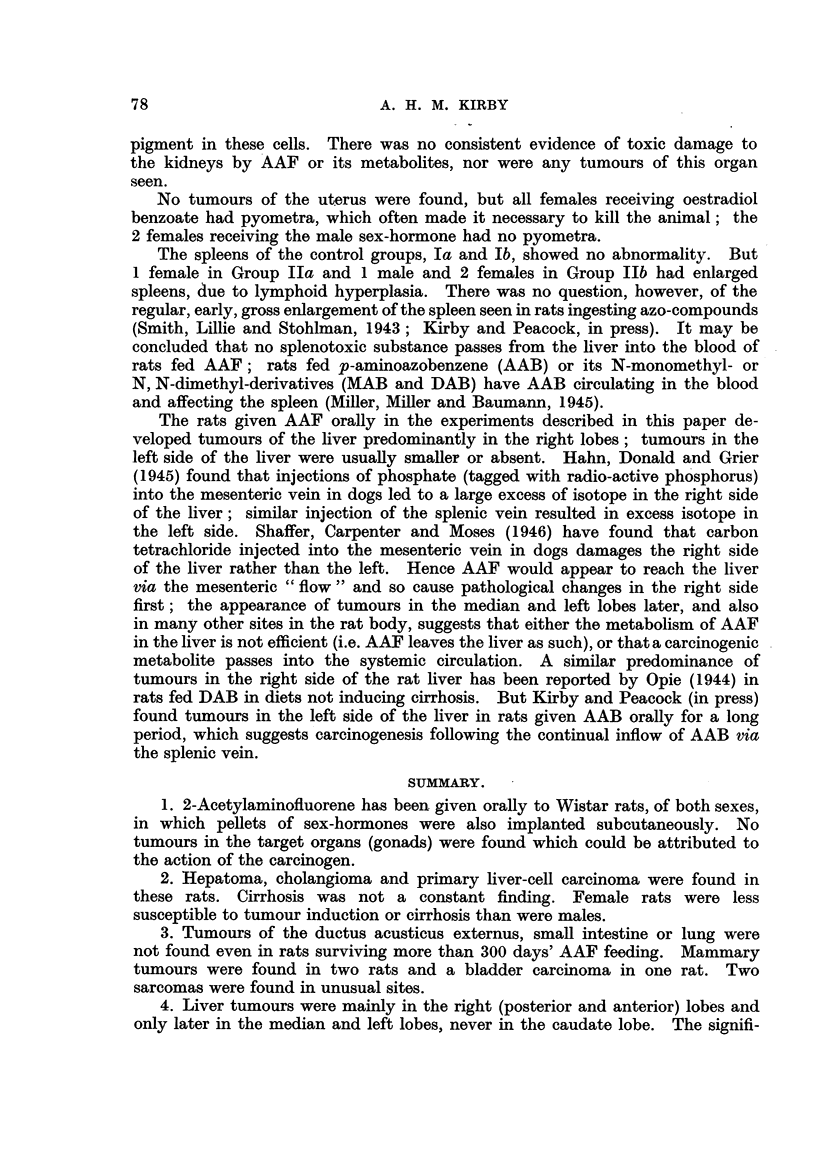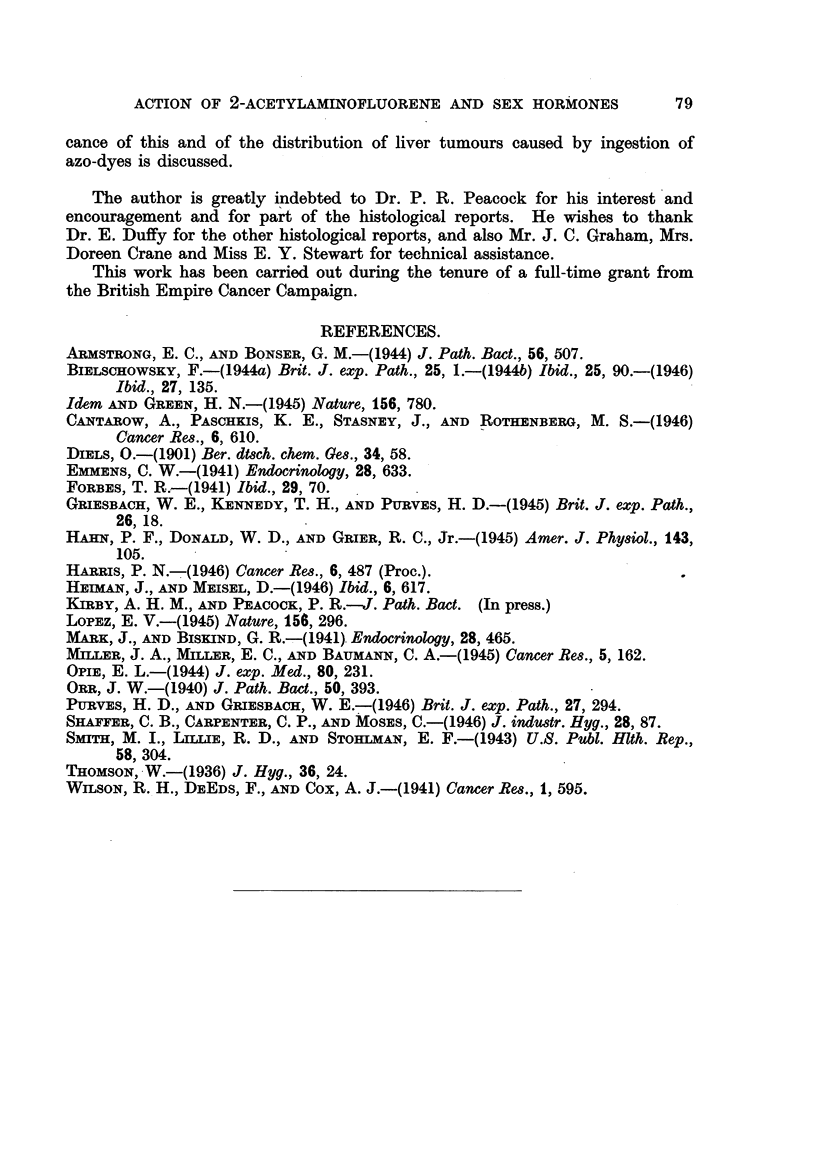# The Combined Action of 2-Acetylaminofluorene and Sex Hormones in the Wistar Rat

**DOI:** 10.1038/bjc.1947.9

**Published:** 1947-03

**Authors:** A. H. M. Kirby


### THE COMBINED ACTION OF 2-ACETYLAMINOFLUORENE AND SEX HORMONES IN THE WISTAR RAT.

A. H. M. KIRBY.

*From the Research Department, Glasgow Royal Cancer Hospital.*

Received for publication February 8, 1947.

THE exceptional interest of 2-acetylaminofluorene (AAF) as a carcinogen was clearly revealed by Wilson, DeEds and Cox (1941), who fed it to inbred albino rats in which only benign mammary tumours had been observed, and found carcinomas of the liver, breast, bladder, ureter, renal pelvis, colon, pancreas, lung and skin, a myogenic sarcoma, and in two cases liver lesions resembling leukaemic infiltration. The results of Bielschowsky (1944 *a* and *b*) and other workers are summarized later in this paper. In CBA mice, neoplasms of liver and uterus (one a sarcoma) and of the urinary bladder were found by Armstrong and Bonser (1944). Bielschowsky and Green (1945) reported a carcinoma of the kidney in a Rhode Island Red cock which had been fed AAF for 45 weeks.

The widespread variety of organs affected by AAF led Bielschowsky (1944*b*) to try to induce tumours of the goitrous thyroid gland with this substance. Solid, parenchymatous goitre was induced by allyl thiourea; in conjunction with AAF, also orally administered, allyl-thiourea induced adenoma in 9 out of 10 rats, while malignancy was observed in 3 of these rats. Griesbach, Kennedy and Purves (1945) claim that the goitrogenic agent in *Brassica* seeds can cause adenoma of the thyroid if given orally for long enough, but only 1 of 8 Wistar rats developed such a lesion. Subsequently Purves and Griesbach (1946) obtained malignant changes in 2 out of 30 Wistar rats given thiourea in their drinking water for nearly two years. Purves and his co-workers consider that the effect of the goitrogens is indirect, operating by inducing hypersecretion of the thyrotropic pituitary hormone. They also suggest that the earlier appearance of malignancy in Bielschowsky's material is due to a synergistic action of AAF and the goitrogen employed, i.e. AAF has no specific action even on adenomatous thyroid tissue.

The success achieved by Bielschowsky in at least accelerating malignant changes in the thyroid of rats by feeding AAF suggested that other organs apparently untouched by AAF alone might undergo neoplastic changes when stimulated to proliferate by suitable means. The sex-organs of the rat can be caused to hypertrophy by the administration of the appropriate sex-hormone, and it was thought that these organs, thus caused to proliferate, might proceed to malignant changes when influenced by orally administered AAF. An exploratory experiment was therefore carried out and, although it failed in its primary objective, the recent publication of a paper by Cantarow, Paschkis, Stasney and Rothenberg (1946) reporting similar work in a different strain of rat makes it desirable to record the results obtained in this laboratory.

### EXPERIMENTAL.

The rats used were of a Wistar strain inbred in this laboratory from rats originally bought from Glaxo, Ltd.; the ages varied from 5 to 22 weeks at the commencement of the experiment. The basal diet for all groups was rat-cake made to the formula of Thomson (1936). 2-Aminofluorene was synthesized from fluorene by the method of Diels (1901),* and acetylated as follows. Up to 1 per cent of concentrated sulphuric acid was added to 2-aminofluorene stirring in an excess of acetic anhydride which became very hot and dissolved the amine. After chilling thoroughly the acetylated amine was filtered on a sintered-glass funnel, and dissolved in hot ethanol. Sufficient water was added to cause permanent turbidity and the solution well cooled. The precipitate, after drying, had m.p. 193–194° C. It was recrystallized from methanol; m.p. 194° C.

AAF was administered in the rat-cake, which was supplied as a fine powder; assuming that a rat would eat 10 g. rat-cake powder per day, AAF was added at a level of 0·04 per cent, i.e. 4 mg. per rat per day.

*Administration of sex-hormones.*—Two sex-hormones were used, testosterone propionate obtained from Boots, Ltd., and oestradiol benzoate obtained from British Drug Houses, Ltd.

Mark and Biskind (1941) found that the subcutaneous implantation of pellets of testosterone propionate in normal rats led to enlargement of both prostates and seminal vesicles. Emmens (1941) found that 40 mg. pellets of disc-shape allowed an absorption of this hormone of 350$\gamma$ per day. Forbes (1941), using 10 mg. pellets, found 90 per cent absorption in 61 days, i.e. an average of 150 $\gamma$ per day. On the basis of these findings it was decided at first to implant one 10 mg. pellet in the flank of each rat every two months. However, in a private communication, Dr. C. W. Emmens pointed out that absorption during the second month would be much reduced, and recommended the use of 20 mg. pellets. Therefore thin disc-shaped pellets weighing 20 mg. were prepared and inserted subcutaneously (after incision and followed by two stitches) in alternate flanks, at intervals of two months. Pellets were completely absorbed by the time another was inserted in the same flank, i.e. by four months. The female hormone, oestradiol benzoate, is known to produce its action in lower concentrations than is required of the male hormone used; pellets were therefore prepared weighing 10 mg., and were inserted in other rats in the same way and at the same intervals.

*Groupings.*—A number of possibilities had to be covered in these experiments; in this way the following groups were determined :—

I. Sex-hormones alone :
- (*a*) Testosterone propionate in virgin males.
  Oestradiol benzoate in virgin females.
- (*b*) Testosterone propionate in mated males.
  Oestradiol benzoate in mated females.

II. Sex-hormones in conjunction with AAF :
- (*a*) Testosterone propionate in virgin males.
  Oestradiol benzoate in virgin females.

* I am indebted to Dr. E. de Barry Barnett for a gift of fluorene; later preparations have been made from fluorene obtained from British Drug Houses, Ltd.

(*b*) Testosterone propionate in mated males.
Oestradiol benzoate in mated females.
(*c*) Testosterone propionate in mated females.
Oestradiol benzoate in mated males.

The number of animals used for each group is recorded in Table I, which

TABLE I.—*Distribution of Rats by Cages.*

| Cage No. | Testosterone propionate. | Testosterone propionate + AAF.* | | Oestradiol benzoate. | Oestradiol benzoate + AAF.* | | Totals. Male. | Totals. Female. |
|---|---|---|---|---|---|---|---|---|
| 85 | .. | 3 M. | | .. | .. | | 3 | .. |
| 86 | 3 M. | .. | | .. | .. | | 3 | .. |
| 87 | .. | 2 M. | 2 F. | .. | .. | | 2 | 2 |
| 88 | 2 M. | .. | | 2 F. | .. | | 2 | 2 |
| 89 | .. | 2 M. | | .. | 2 F. | | 2 | 2 |
| 90 | .. | .. | | 4 F. | .. | | .. | 4 |
| 91 | .. | .. | | .. | 3 F. | | .. | 3 |
| 92 | .. | .. | | .. | 2 M. | 2 F. | 2 | 2 |
| Totals | 5 M. | 7 M. | 2 F. | 6 F. | 2 M. | 7 F. | 14 | 15 |

* AAF = 2-acetylaminofluorene.

also shows the distribution. Full data for individual rats are recorded in Table II.

The average weights of liver, prostates, testes + epididymis and seminal vesicles in normal adult males of our strain are as follows :—

Liver : 4 per cent of body weight.
Prostates : 0·15 per cent of body weight.
Testes + epididymis : 2 per cent of body weight.
Seminal vesicles : 0·3 per cent of body weight.

### RESULTS.

The incidence of tumours in each group is summarized in Table III. A spindle-cell sarcoma of the left seminal vesicle was found in rat 354 (Group II*b*), and a whorled, spindle-cell sarcoma was found lying above the liver of rat 339 (Group II*a*). Tapeworms were not present in the liver of any rat.

### DISCUSSION.

The results presented here agree with those of Cantarow *et al.* (1946) in that no carcinomas were induced in hypertrophied gonads in either male or female rats. The reason for this may well be that suggested by these workers, namely that " the hyperplasia of target organs of sex-hormones is a functioning hyperplasia, whereas that induced in the thyroid by goitrogenic agents is non-functioning." Thus the type of hyperplasia induced by Bielschowsky in the rat thyroid in which growth is accompanied by loss of differentiation would appear to have been a suitable basis for carcinogenesis, whereas the hyperplasia associated with increased differentiation induced in sex organs by over-stimulation with sex-hormones is apparently unsuitable. The reparative hyperplasia found in the livers of rats given N, N-dimethyl-*p*-aminoazobenzene (butter yellow) is often accompanied by primary liver-cell carcinoma, and it has been considered that the carcinoma arises in such nodules of regenerative tissue (Orr, 1940 ; see also

TABLE II.—*Data for Individual Rats.*

| Group. | Rat No. | Sex. | Age at start, in weeks. | Nature of pellets inserted. | Pellets inserted on days: | Experimental period in days. | Weight at death. (g.) | § Relative Weights (%) of Prostates. | Testes + epididymis. | Seminal vesicles. | Liver. |
|---|---|---|---|---|---|---|---|---|---|---|---|
| I*a* | 341 | M. | 9 | 20 mg. T.P. | 1, 84, 149, 210, 273 | K.‖ 370 | 255 | 0·5 | 0·8 | .. | 4·5 |
| | 342 | M. | 6 | ,, | 1, 84, 149, 210, 273 | K. 370 | 290 | 0·4 | 0·8 | 0·6 | 5·3 |
| | 343 | M. | 6 | ,, | 1, 62, 127, 188, 251 | K. 348 | 270 | 0·5 | 0·9 | 0·7 | 4·8 |
| | 357 | F. | 14 | 10 mg. E.B. | 85, 149 | 255 | 190 | .. | .. | .. | .. |
| | 358 | F. | 14 | ,, | 85, 149, 210 | 350 | 135 | .. | .. | .. | .. |
| | 359 | F. | 14 | ,, | 85, 149 | K. 232 | 195 | .. | .. | .. | .. |
| | 360 | F. | 5 | ,, | 1, 52, 113 | 272 | 130 | .. | .. | .. | .. |
| I*b* | 349 | M. | 10 | 20 mg. T.P. | 1, 84, 149, 210, 273 | K. 373 | 275 | 0·4 | 0·8 | 0·5 | 6·0 |
| | 350 | M. | 10 | ,, | 1, 84, 149, 210, 273 | K. 369 | 310 | 0·4 | 0·8 | 0·5 | 4·3 |
| | 351 | F. | 10* | 10 mg. E.B. | 85, 149 | 180 | 170 | .. | .. | .. | .. |
| | 352 | F. | 14 | ,, | 85 | Lost by cannibalism | .. | .. | .. | .. | .. |
| II*a* | 337 | M. | 14 | 20 mg. T.P. | 1, 84, 149, 210, 273 | K. 366 | 200 | 0·4 | 1·2 | 0·8 | 17·0 |
| | 338 | M. | 10 | ,, | 1, 84, 149, 210, 273 | K. 367 | 280 | 0·3 | 1·1 | 0·3 | .. |
| | 339 | M. | 12 | ,, | 1, 84, 149, 210, 273 | K. 367 | 240 | 0·4 | 0·5 | 0·6 | 13·3 |
| | 361 | F. | 22 | 10 mg. E.B. | 17, 81 | 180 | 170 | .. | .. | .. | .. |
| | 362 | F. | 22 | ,, | 17, 81, 142 | K. 301 | 145 | .. | .. | .. | .. |
| | 363 | F. | 12 | ,, | 17, 81 | K. 203 | 180 | .. | .. | .. | .. |
| II*b* | 345 | M. | 6 | 20 mg. T.P. | 1, 62, 127, 188, 251 | K. 319 | 225 | .. | .. | .. | .. |
| | 348 | M. | 12 | ,, | 1, 84, 149, 210, 273 | K. 372 | 235 | 0·4 | 0·7 | 0·4 | 19·0 |
| | 353 | M. | 9 | ,, | 1, 84, 149, 210 | K. 377 | 240 | 0·4 | 1·4 | .. | 16·0 |
| | 354 | M. | 6 | ,, | 1, 84, 149, 210, 273 | 377 | 250 | .. | .. | .. | 14·6 |
| | 355 | F. | 9† | 10 mg. E.B. | 85 | K. 141 | 155 | .. | .. | .. | .. |
| | 356 | F. | 14‡ | ,, | 149 | K. 378 | 180 | .. | .. | .. | 18·4 |
| | 367 | F. | 5 | ,, | 1, 52, 113 | K. 238 | 165 | .. | .. | .. | .. |
| | 368 | F. | 5 | ,, | 1, 52, 113 | K. 238 | 140 | .. | .. | .. | .. |
| II*c* | 365 | M. | 5 | 10 mg. E.B. | 1, 52, 113 | K. 280 | 130 | Not visible | 0·2 | 0·15 | 11·1 |
| | 366 | M. | 5 | ,, | 1, 52, 113 | K. 276 | 115 | .. | .. | 0·2 | 7·3 |
| | 346 | F. | 14 | 5 mg. T.P. in oil | 1, 15, 29, 43 | K. 82 | 180 | .. | .. | .. | .. |
| | 347 | F. | 12 | 20 mg. T.P. | 1, 84, 149, 210, 273 | K. 372 | 210 | .. | .. | .. | 16·0 |

T.P. Testosterone propionate.

E.B. Oestradiol benzoate.

* Litter at 18 weeks.

† Litter at 20 weeks.

‡ Litters at 26 and 38 weeks.

§ Expressed as percentage of total weight at death.

‖ K. = killed.

TABLE III.—*Summary of Tumours Found in Each Group.*

| Group. | Number of rats. M. | Number of rats. F. | Site. Liver. | Mammary glands. | Bladder. | Lung. | Stomach. |
|---|---|---|---|---|---|---|---|
| I*a* | 3 | .. | None | None | None | None | None |
| | | 4 | " | 1 mixed tumour* | " | 1 undifferentiated bronchial carcinoma | " |
| I*b* | 2 | 2 | " | None | " | None | " |
| II*a* | 3 | .. | Hepatomas in all 3 | " | " | " | " |
| | | 3† | Hepatomas in both | 1, undifferentiated adenocarcinoma in right caudal gland | " | " | 1, ulceropapillomas in fore-stomach |
| II*b* | 4 | .. | 3, hepatomas; 1, carcinoma; 1, cholangiomas | None | " | " | None |
| | | 4‡ | 2, hepatomas and cholangiomas | 1, undifferentiated adenocarcinoma in left axillary gland | " | " | 1, ulceropapillomas in fore-stomach |
| II*c* | 2 | .. | Hepatomas in both | 1, lactation in two lobes | 1, squamous carcinoma | " | None |
| | | 1§ | Hepatoma and cholangioma | None | None | " | " |

* Adenoacanthoma and intra-canalicular adenomas.

† 1 died at 180 days with advanced post-mortem changes but no visible tumours.

‡ 1 killed at 141 days showing regenerative hyperplasia of liver.

§ 1 killed at 82 days showing no abnormality.

Opie, 1944). If this sequence is true, then the hyperplastic liver tissue may be more susceptible than resting liver tissue to the carcinogenic action of azo dyes. Preliminary work reported here suggests that the initial stage of reaction to AAF is not degenerative, but that the hyperplasia is induced by the compound directly.

A summary of results obtained by workers with AAF in rats is provided in Table IV. From this it is evident that AAF can induce tumours, usually malignant, in an even greater variety of sites than was apparent from the work of Wilson *et al.* (1941). Wilson *et al.* illustrate a " carcinoma arising adjacent to the external auditory canal," and discuss the histogenesis of this and other tumours in this locality included by them under "subcutaneous tumours." Bielschowsky first drew attention to tumours of the ductus acusticus externus as a separate entity. No such tumours were seen in any of the rats in the experiments described in this paper, although 9 survived more than 300 days' administration of AAF. As Wistar rats were also used by Bielschowsky, who found these tumours in 18/93 rats given AAF orally up to 210 days, the influence of diet seems to have some importance. When allyl-thiourea was also given, the incidence of tumours rose to 6/10. Lopez (1945) also found external meatus tumours in 2/4 rats. On the other hand, Heiman and Meisel (1946) make no mention of such tumours among 39 Wistar rats given AAF orally for up to 227 days. Bielschowsky (1944*a*) seems to have used a simpler and, perhaps, less adequate diet than did Heiman and Meisel; the basal diet used in our experiments was also better balanced than that of Bielschowsky. It has been reported (Harris, 1946) that protection against liver tumours due to AAF could not be obtained by adding to the basal diet supplements of foodstuffs known to afford considerable protection to the rat liver against carcinogenesis by azo-dyes. This further supports the apparent difference in carcinogenesis between azo-dyes and AAF, the former being dependent on reparative hyperplasia as a precancerous stage. It is significant, however, that Heiman and Meisel (1946) found that Wistar rats fed AAF plus aromatic amino-acids were free from liver tumours and cirrhosis up to 227 days. This may indicate that, in the rat liver, the carcinogenic process in the case of AAF depends on the " blocking " of a normal metabolic mechanism dependent on adequate supplies of aromatic amino-acids rather than on sulphur-amino-acids and/or riboflavin, which appear to be needed in excess of normal requirements to prevent (or delay) carcinogenesis of the liver due to azo-dyes.

Other sites in the Wistar rat in which Bielschowsky found tumours following administration of AAF include the small intestine (5/93 cases); Heiman and Meisel report no tumours in this site, and none was found in our experiments. Moreover, Bielschowsky reports mammary tumours in 27/93 Wistar rats, including 3 males; Heiman and Meisel found them in only 2/39 rats. It has been shown by Mark and Biskind (1941) that adeno-carcinoma of the breast in rats may follow the implantation of oestradiol benzoate pellets without administration of any other carcinogen. In our series, 1 of 3 control rats receiving oestradiol benzoate and 2/8 females receiving AAF as well developed mammary tumours; 1 male in Group II*c* (oestradiol benzoate pellets) had lactating glands but no tumours. Hence the action of AAF on proliferating mammary tissue was only slight in our experiments.

In the matter of liver tumours, our series has yielded results in full accordance

### Table IV.

| Author(s). | Rat type. | Number of rats. | Periods up to— | Level AAF. | Tumour sites. Liver. M. | F. | Mammary tissue. M. | F. | Bladder. M. | F. | External ear. M. | F. | Small intestine. M. | F. | Large intestine. M. | F. |
|---|---|---|---|---|---|---|---|---|---|---|---|---|---|---|---|---|
| Wilson *et al.* (1941) | Inbred albino | 39 | 95 days or more | 0·031% | 8/9 | | 1/9 | | 4/9 | | .. | .. | .. | .. | .. | .. |
| | | | | 0·062% | 5/5 | | 1/5 | | 2/5 | | .. | .. | .. | .. | .. | .. |
| | | | | 0·125% | 23/25 | | 1/25 | | 19/25 | | .. | .. | .. | .. | 1/25 | |
| Bielschowsky (1944*a*) | Wistar | 104 | 210 days | 0·04% | 39/46 | 16/47 | 3/46 | 24/47 | .. | .. | 10/46 | 8/47 | 3/46 | 2/47 | .. | .. |
| Bielschowsky (1944*b*) | ,, | 10 | 210 ,, | 0·04% + 0·06% allyl-thiourea | 4/5 | 1/5 | 0/5 | 2/5 | .. | .. | 2/5 | 4/5 | .. | .. | .. | .. |
| Lopez (1945) | ? | 4 | 168 ,, | 0·05% | 2/4 | | 2/4 | | .. | .. | 2/4 | | .. | .. | .. | .. |
| Bielschowsky (1946) | Piebald | 38 | 293 ,, | 0·04% | 10/13 | 10/25 | .. | 1/25 | .. | .. | 10/13 | 20/25 | 6/13 | 14/25 | .. | 2/25 |
| Cantarow *et al.* (1946) | Sherman | 21 controls | 316 ,, | 0·03% | 2/3 | 4/18 | .. | .. | .. | .. | .. | .. | .. | .. | .. | .. |
| | | 15 | 316 ,, | 0·03% + 0·05% thiouracil | 0/6 | 0/9 | .. | .. | .. | .. | .. | .. | .. | .. | .. | .. |
| | | 25 F. oestrogen | 300 ,, | 0·03% | .. | 13/25 | .. | .. | .. | .. | .. | .. | .. | .. | .. | .. |
| | | 15 M. androgen | 302 ,, | 0·03% | 13/15 | .. | .. | .. | .. | .. | .. | .. | .. | .. | .. | .. |
| Heiman and Meisel (1946) | Wistar | 39 | 227 ,, | 5 mg. per day | 1/39 | | 2/39 | | .. | .. | .. | .. | .. | .. | .. | .. |
| Kirby | ,, | 17 | 378 ,, | 0·04% | 9/9 | 5/8 | 0/9 | 2/8 | 1/9 | 0/8 | 0/9 | 0/8 | 0/9 | 0/8 | 0/9 | 0/8 |

TABLE IV (continued).

| Author(s). | Rat type. | Number of rats. | Periods up to— | Level AAF. | Tumour sites. Skin. M. | Skin. F. | Uterus. M. | Uterus. F. | Ureter. M. | Ureter. F. | Kidney. M. | Kidney. F. | Lung. M. | Lung. F. | Brain. M. | Brain. F. |
|---|---|---|---|---|---|---|---|---|---|---|---|---|---|---|---|---|
| Wilson *et al.* (1941) | Inbred albino | 39 | 95 days or more | 0·031% | 4/9 | | .. | .. | .. | .. | 5/9 | | 3/9 | | .. | .. |
| | | | | 0·062% | 0/5 | | .. | .. | .. | .. | 0/5 | | 1/5 | | .. | .. |
| | | | | 0·125% | 4/5 | | .. | .. | 1/25 | | 13/25 | | 9/25 | | .. | .. |
| Bielschowsky (1944*a*) | Wistar | 104 | 210 days | 0·04% | .. | 1/47 | .. | 1/47 | .. | .. | .. | .. | .. | .. | .. | .. |
| Bielschowsky (1944*b*) | ,, | 10 | 210 ,, | 0·04% + 0·06% allyl-thiourea | .. | .. | .. | .. | .. | .. | 1/5 | 0/5 | .. | .. | .. | .. |
| Lopez (1945) | ? | 4 | 168 ,, | 0·05% | .. | .. | .. | .. | .. | .. | .. | .. | .. | .. | 1/4 | |
| Bielschowsky (1946) | Piebald | 38 | 293 ,, | 0·04% | .. | .. | .. | .. | .. | .. | .. | .. | 1/13 | 2/25 | .. | .. |
| Cantarow *et al.* (1946) | Sherman | 21 controls | 316 ,, | 0·03% | .. | .. | .. | .. | .. | .. | .. | .. | .. | .. | .. | .. |
| | | 15 | 316 ,, | 0·03% + 0·05% thiouracil | .. | .. | .. | .. | .. | .. | .. | .. | .. | .. | .. | .. |
| | | 25 F. oestrogen | 300 ,, | 0·03% | .. | .. | .. | .. | .. | .. | .. | .. | .. | .. | .. | .. |
| | | 15 M. androgen | 302 ,, | 0·03% | .. | .. | .. | .. | .. | .. | .. | .. | .. | .. | .. | .. |
| Heiman and Meisel (1946) | Wistar | 39 | 227 ,, | 5 mg. per day | .. | .. | .. | .. | .. | .. | .. | .. | .. | .. | .. | .. |
| Kirby | ,, | 17 | 378 ,, | 0·04% | 0/9 | 0/8 | 0/9 | 0/8 | 0/9 | 0/8 | 0/9 | 0/8 | None | None | None | None |

### Table IV (continued).

| Author(s). | Rat type. | Number of rats. | Periods up to— | Level AAF. | Tumour sites. Sarcoma. M. | Sarcoma. F. | Pancreas. M. | Pancreas. F. | Sub-maxillary gland. | Thyroid. | Para-thyroid. |
|---|---|---|---|---|---|---|---|---|---|---|---|
| Wilson *et al.* (1941) | Inbred albino | 39 | 95 days or more | 0·031% | .. | .. | 5/9 | | .. | .. | .. |
| | | | | 0·062% | .. | .. | 2/5 | | .. | .. | .. |
| | | | | 0·125% | 1/25 | | 11/25 | | .. | .. | .. |
| Bielschowsky (1944*a*) | Wistar | 104 | 210 days | 0·04% | .. | .. | .. | .. | .. | .. | .. |
| Bielschowsky (1944*b*) | ,, | 10 | 210 ,, | 0·04% + 0·06% allyl-thiourea | .. | .. | .. | .. | .. | 9/10 | .. |
| Lopez (1945) | ? | 4 | 168 ,, | 0·05% | .. | .. | .. | .. | .. | .. | .. |
| Bielschowsky (1946) | Piebald | 38 | 293 ,, | 0·04% | .. | .. | .. | .. | .. | .. | .. |
| Cantarow *et al.* (1946) | Sherman | 21 controls | 316 ,, | 0·03% | .. | .. | .. | .. | .. | .. | .. |
| | | 15 | 316 ,, | 0·03% + 0·05% thiouracil | .. | .. | .. | .. | .. | .. | .. |
| | | 25 F. oestrogen | 300 ,, | 0·03% | .. | .. | .. | .. | .. | .. | .. |
| | | 15 M. androgen | 302 ,, | 0·03% | .. | .. | .. | .. | .. | .. | .. |
| Heiman and Meisel (1946) | Wistar | 39 | 227 ,, | 5 mg. per day | 1/39 | | .. | .. | 5/39 | 1/39 | 2/39 |
| Kirby | ,, | 17 | 378 ,, | 0·04% | 2/9 | 0/8 | None | None | None | None | None |

with those of Bielschowsky. All of 9 male rats, dying between 276 and 377 days, had liver tumours ; most had some degree of cirrhosis, and cholangiectasis, in varying degree, was frequently found. One rat, in Group II*b*, showed lesions varying from fatty degeneration to acute yellow atrophy ; cholangioma with haemorrhage into cystic ducts was also present. The other 8 had either diffuse or trabecular hepatoma ; bile-duct proliferation was also seen in these rats. Of the 8 female rats examined, 2 died at 82 and 141 days respectively without tumours ; of the remaining 6, 5 showed hepatoma and 3 cholangioma. Cholangiectasis was present in most, but cirrhosis in only 1 case.

Cantarow *et al.* (1946) remark that " the occurrence of the cystic and neoplastic lesions was not paralleled by degenerative or fibrotic processes in the liver," and this was certainly true of the livers in our series.

Cantarow *et al.* (1946) only report on the liver lesions induced in Sherman rats by orally ingested AAF. Here again, however, it is clear that the incidence is lower in females than in males. The types of liver tumours seem to be essentially the same as in Wistar rats. This is also true of the liver tumours obtained by Bielschowsky in piebald rats, in which the sex-ratio is also maintained and the incidence much the same. In the Sherman strain, Cantarow *et al.* found the incidence of liver tumours in female rats raised when oestradiol dipropionate was injected intramuscularly ; but the complete absence of liver neoplasms in rats given thiouracil was much more striking. The latter group ate less of the AAF diet than did controls receiving no thiouracil, but more than the oestrogenized rats.

The incidence of mammary tumours in piebald rats was very low compared with that in Wistar rats on the same diet (Bielschowsky, 1946). But the incidence of tumours of the ductus acousticus externus was much higher in the piebald rats, in which tumours of the small intestine were also much more frequent. Moreover, lung tumours were found in this strain, but have not been reported in Wistar rats given AAF and were not found in our series, although one control female (Group I*a*) was found to have undifferentiated bronchial carcinoma.

The albino rats of Wilson *et al.* (1941) resemble the piebald strain in the low incidence of mammary tumours, but differ in the absence of tumours of the small intestine. On the other hand, lung and liver tumours were rather more frequent. Moreover, Wilson *et al.* found tumours in the urinary system (kidney, ureter and bladder) and in the pancreas. The latter have not been found in any other strain of rat, and tumours of the kidney and bladder only once each.

It would seem that, if sufficient strains of rat were employed, possibly no site in the body would be found immune from the carcinogenic action of AAF. In the strains so far tested, only mammary tissue among the sex-organs seems to be susceptible, and this tissue is liable to develop carcinoma due to the action of oestrogens alone. Sarcomas are rare, and are not induced by subcutaneous injection of AAF, at least in Wistar rats. In our series, a whorled, spindle-cell sarcoma was found in the omentum covering the left lateral lobe of the liver in one rat, and a spindle-cell sarcoma was present in the left seminal vesicle of another.

It remains to discuss a few other points of interest. Toxic damage to kidneys was seen in some animals of all groups ; rats receiving oestradiol benzoate pellets, with or without AAF, tended to show degeneration of the epithelium of the first and second convoluted tubules with the presence of a dark yellow pigment in these cells. There was no consistent evidence of toxic damage to the kidneys by AAF or its metabolites, nor were any tumours of this organ seen.

No tumours of the uterus were found, but all females receiving oestradiol benzoate had pyometra, which often made it necessary to kill the animal; the 2 females receiving the male sex-hormone had no pyometra.

The spleens of the control groups, I*a* and I*b*, showed no abnormality. But 1 female in Group II*a* and 1 male and 2 females in Group II*b* had enlarged spleens, due to lymphoid hyperplasia. There was no question, however, of the regular, early, gross enlargement of the spleen seen in rats ingesting azo-compounds (Smith, Lillie and Stohlman, 1943; Kirby and Peacock, in press). It may be concluded that no splenotoxic substance passes from the liver into the blood of rats fed AAF; rats fed *p*-aminoazobenzene (AAB) or its N-monomethyl- or N, N-dimethyl-derivatives (MAB and DAB) have AAB circulating in the blood and affecting the spleen (Miller, Miller and Baumann, 1945).

The rats given AAF orally in the experiments described in this paper developed tumours of the liver predominantly in the right lobes; tumours in the left side of the liver were usually smaller or absent. Hahn, Donald and Grier (1945) found that injections of phosphate (tagged with radio-active phosphorus) into the mesenteric vein in dogs led to a large excess of isotope in the right side of the liver; similar injection of the splenic vein resulted in excess isotope in the left side. Shaffer, Carpenter and Moses (1946) have found that carbon tetrachloride injected into the mesenteric vein in dogs damages the right side of the liver rather than the left. Hence AAF would appear to reach the liver *via* the mesenteric "flow" and so cause pathological changes in the right side first; the appearance of tumours in the median and left lobes later, and also in many other sites in the rat body, suggests that either the metabolism of AAF in the liver is not efficient (i.e. AAF leaves the liver as such), or that a carcinogenic metabolite passes into the systemic circulation. A similar predominance of tumours in the right side of the rat liver has been reported by Opie (1944) in rats fed DAB in diets not inducing cirrhosis. But Kirby and Peacock (in press) found tumours in the left side of the liver in rats given AAB orally for a long period, which suggests carcinogenesis following the continual inflow of AAB *via* the splenic vein.

### SUMMARY.

1. 2-Acetylaminofluorene has been given orally to Wistar rats, of both sexes, in which pellets of sex-hormones were also implanted subcutaneously. No tumours in the target organs (gonads) were found which could be attributed to the action of the carcinogen.

2. Hepatoma, cholangioma and primary liver-cell carcinoma were found in these rats. Cirrhosis was not a constant finding. Female rats were less susceptible to tumour induction or cirrhosis than were males.

3. Tumours of the ductus acusticus externus, small intestine or lung were not found even in rats surviving more than 300 days' AAF feeding. Mammary tumours were found in two rats and a bladder carcinoma in one rat. Two sarcomas were found in unusual sites.

4. Liver tumours were mainly in the right (posterior and anterior) lobes and only later in the median and left lobes, never in the caudate lobe. The significance of this and of the distribution of liver tumours caused by ingestion of azo-dyes is discussed.

The author is greatly indebted to Dr. P. R. Peacock for his interest and encouragement and for part of the histological reports. He wishes to thank Dr. E. Duffy for the other histological reports, and also Mr. J. C. Graham, Mrs. Doreen Crane and Miss E. Y. Stewart for technical assistance.

This work has been carried out during the tenure of a full-time grant from the British Empire Cancer Campaign.

### REFERENCES.

Armstrong, E. C., and Bonser, G. M.—(1944) *J. Path. Bact.*, **56**, 507.
Bielschowsky, F.—(1944*a*) *Brit. J. exp. Path.*, **25**, 1.—(1944*b*) *Ibid.*, **25**, 90.—(1946) *Ibid.*, **27**, 135.
*Idem* and Green, H. N.—(1945) *Nature*, **156**, 780.
Cantarow, A., Paschkis, K. E., Stasney, J., and Rothenberg, M. S.—(1946) *Cancer Res.*, **6**, 610.
Diels, O.—(1901) *Ber. dtsch. chem. Ges.*, **34**, 58.
Emmens, C. W.—(1941) *Endocrinology*, **28**, 633.
Forbes, T. R.—(1941) *Ibid.*, **29**, 70.
Griesbach, W. E., Kennedy, T. H., and Purves, H. D.—(1945) *Brit. J. exp. Path.*, **26**, 18.
Hahn, P. F., Donald, W. D., and Grier, R. C., Jr.—(1945) *Amer. J. Physiol.*, **143**, 105.
Harris, P. N.—(1946) *Cancer Res.*, **6**, 487 (Proc.).
Heiman, J., and Meisel, D.—(1946) *Ibid.*, **6**, 617.
Kirby, A. H. M., and Peacock, P. R.—*J. Path. Bact.* (In press.)
Lopez, E. V.—(1945) *Nature*, **156**, 296.
Mark, J., and Biskind, G. R.—(1941) *Endocrinology*, **28**, 465.
Miller, J. A., Miller, E. C., and Baumann, C. A.—(1945) *Cancer Res.*, **5**, 162.
Opie, E. L.—(1944) *J. exp. Med.*, **80**, 231.
Orr, J. W.—(1940) *J. Path. Bact.*, **50**, 393.
Purves, H. D., and Griesbach, W. E.—(1946) *Brit. J. exp. Path.*, **27**, 294.
Shaffer, C. B., Carpenter, C. P., and Moses, C.—(1946) *J. industr. Hyg.*, **28**, 87.
Smith, M. I., Lillie, R. D., and Stohlman, E. F.—(1943) *U.S. Publ. Hlth. Rep.*, **58**, 304.
Thomson, W.—(1936) *J. Hyg.*, **36**, 24.
Wilson, R. H., DeEds, F., and Cox, A. J.—(1941) *Cancer Res.*, **1**, 595.